# Supplementary material for: CD8+ T cell self-tolerance permits responsiveness but limits tissue damage
Source: eLife. 2021 Apr 30;10:e65615. doi: 10.7554/eLife.65615 (PMC8147182; doi:10.7554/eLife.65615)
Supplement: Figure 1—source data 2. [file elife-65615-fig1-data2.docx]

**Table 1A.** Primers used to screen prospective clones for homologous recombination of the *Dct* knockout construct

| **Position** | **Forward Primer** | **Sequence (5’ to 3’)** | **Reverse Primer** | **Sequences (5’ to 3’)** | **Product Size (bp)** |
| --- | --- | --- | --- | --- | --- |
|  |  |  |  |  |  |
| 5' External | S-Arm-SF | GCA GCT CCA GGT GTG CCA CTT | Linker-R1 | GTT AGC CAG AGG GCC GCT CT | 4007 |
| 3' External | Neo 9F | AAG GGG CCA CCA AAG AAC GG | 3-Arm-4R | GCC ACT TCG CAG CCA GACGA | 5286 |
| 3' Internal | Neo-4F | CTA AAG CGCATG CTC CAG ACT GCC | 3-Arm-In-2R | CTT CCG GGA GAG GCT CTG GCT | 3883 |
| 5' Internal | 5-Arm-In-F | CTA AAG CGCATG CTC CAG ACT GCC | Linker-R1 | GTT AGC CAG AGG GCC GCT CT | 2907 |
