# Supplementary material for: CD8+ T cell self-tolerance permits responsiveness but limits tissue damage
Source: eLife. 2021 Apr 30;10:e65615. doi: 10.7554/eLife.65615 (PMC8147182; doi:10.7554/eLife.65615)
Supplement: Figure 1—source data 3. [file elife-65615-fig1-data3.docx]

**Table 1B.** Primers used to screen mice to confirm deletion of the *Dct* gene

| **Position** | **Forward Primer** | **Sequence (5’ to 3’)** | **Reverse Primer** | **Sequences (5’ to 3’)** | **Product Size (bp)** |
| --- | --- | --- | --- | --- | --- |
|  |  |  |  |  |  |
| Exon 1 | Dct-Ex1-F | TCC CAA TTA AGA AGG CAT GG | Dct-Ex1-R | CCA GGG TCT GGT GTC TGT TT | 294 |
| Exon 2 | Dct-Ex2-F | CCC GAC TGT AAT CGG AAG AA | Dct-Ex2-R | TTG CGT GGT GAT CAC GTA GT | 299 |
| Exon 3 | Dct-Ex3-F | TAC CAT CTG TTG TGG CTG GA | Dct-Ex3-R | AAA AGC ATC CAC ACT GGG TC | 100 |
| Exon 4 | Dct-Ex4-F | CAG TTT TCC TAT GGG AGG CA | Dct-Ex4-R | GGT AGG GTT GAT ACT CGC CA | 166 |
| Exon 5 | Dct-Ex5-F | TGA GAC CCT TTC TCG CAG TT | Dct-Ex5-R | GTG TCT GCC AAA ACC TGG AT | 179 |
| Exon 6 | Dct-Ex6-F | GTG GCA TAG CCT CTG CTT TC | Dct-Ex6-R | AGT CCA GTG TTC CGT CTG CT | 135 |
| Exon 7 | Dct-Ex7-F | TCT TGC AGG TCC TCC ACT CT | Dct-Ex7-R | AAG TTG CTC TGC GGT TAG GA | 201 |
